# Homing Peptide-Based Targeting of Tenascin-C and Fibronectin in Endometriosis

**DOI:** 10.3390/nano11123257

**Published:** 2021-11-30

**Authors:** Lorena Simón-Gracia, Kristina Kiisholts, Vilma Petrikaitė, Allan Tobi, Merli Saare, Prakash Lingasamy, Maire Peters, Andres Salumets, Tambet Teesalu

**Affiliations:** 1 Laboratory of Precision and Nanomedicine, Department of Biomedicine and Translational Medicine, University of Tartu, 50411 Tartu, Estonia; Lorena.Simon.Gracia@ut.ee (L.S.-G.); allan.tobi@ut.ee (A.T.); prakash.lingasamy@ut.ee (P.L.); 2 Competence Centre on Health Technologies, 50411 Tartu, Estonia; kristina.kiisholts@ut.ee (K.K.); merli.saare@ut.ee (M.S.); maire.peters@ut.ee (M.P.); Andres.Salumets@ut.ee (A.S.); 3 Laboratory of Drug Target Histopathology, Institute of Cardiology, Lithuanian University of Health Sciences, 44307 Kaunas, Lithuania; vilmapetrikaite@gmail.com; 4 Life Sciences Center, Institute of Biotechnology, Vilnius University, 10257 Vilnius, Lithuania; 5 Department of Obstetrics and Gynecology, Institute of Clinical Medicine, University of Tartu, 50406 Tartu, Estonia; 6 Institute of Genomics, University of Tartu, 51010 Tartu, Estonia; 7 Division of Obstetrics and Gynecology, Department of Clinical Science, Intervention and Technology (CLINTEC), Karolinska Institutet, 14152 Stockholm, Sweden; 8 Center for Nanomedicine, Department of Cell, Molecular and Developmental Biology, University of California at Santa Barbara, Santa Barbara, CA 93106, USA

**Keywords:** endometriosis, homing peptide, silver nanoparticles, nanomedicine, extracellular matrix

## Abstract

The current diagnostic and therapeutic strategies for endometriosis are limited. Although endometriosis is a benign condition, some of its traits, such as increased cell invasion, migration, tissue inflammation, and angiogenesis are similar to cancer. Here we explored the application of homing peptides for precision delivery of diagnostic and therapeutic compounds to endometriotic lesions. First, we audited a panel of peptide phages for the binding to the cultured immortalized endometriotic epithelial 12Z and eutopic stromal HESC cell lines. The bacteriophages displaying PL1 peptide that engages with angiogenic extracellular matrix overexpressed in solid tumors showed the strongest binding to both cell lines. The receptors of PL1 peptide, tenascin C domain C (TNC-C) and fibronectin Extra Domain-B (Fn-EDB), were expressed in both cells. Silver nanoparticles functionalized with synthetic PL1 peptide showed specific internalization in 12Z and HESC cells. Treatment with PL1-nanoparticles loaded with the potent antimitotic drug monomethyl auristatin E decreased the viability of endometriotic cells in 2D and 3D cultures. Finally, PL1-nanoparticless bound to the cryosections of clinical peritoneal endometriotic lesions in the areas positive for TNC-C and Fn-EDB immunoreactivities and not to sections of normal endometrium. Our findings suggest potential applications for PL1-guided nanoparticles in precision diagnosis and therapy of endometriosis.

## 1. Introduction

In endometriosis, the endometrial glandular epithelial and stromal cells establish outgrowths outside the uterine tissue throughout the abdominal–pelvic peritoneum and visceral organs [1]. Endometriosis affects ~5–10% of women in reproductive age, and it is a frequent source of infertility and chronic pelvic pain [2]. Available treatments (painkillers, laparoscopic resection of endometriotic lesions, and hormonal treatments such as contraceptives, gonadotropin releasing hormone agonists (GnRHa), and antagonists) only alleviate the symptoms of the disease and/or are associated with serious side effects [3]. For example, hormonal treatments are effective in pain reduction but cause frequent hyperandrogenic side effects and are not suitable if the woman desires pregnancy [3]. In cases where patients are refractory to treatment with oral contraceptives, progestins, GnRHa, and aromatase inhibitors are used, but these therapies have a high rate of adverse effects [4]. Although laparoscopy can temporarily alleviate the symptoms, complete excision of the lesions remains challenging, leading to recurrence over time [5].

Despite its benign clinical behavior and normal-appearing histological features, endometriosis shares some characteristics with malignant processes, such as increased cell proliferation, migration, invasion and resistance to apoptosis [6]. Some mutations present in malignant neoplasms are also found in endometriotic lesions [7]. Moreover, some extracellular matrix (ECM) proteins upregulated in solid tumors are overexpressed in ectopic endometrium. ECM is involved in the modulation of cellular adhesion and invasion, and aberrant cell-ECM interactions may be functionally involved in the progression of endometriosis [8]. For example, the ECM molecule tenascin C (TNC) and its spliced variants play an important role in neovascularization, modulation of cell adhesion, and cell migration [9]. In endometriotic lesions, the expression of TNC is upregulated and, unlike in eutopic endometrium, TNC expression in the lesions is not dependent on the menstrual cycle [10,11]. Spliced isoforms of fibronectin are also overexpressed in human endometriotic lesions [12]. Expression of alternatively spliced fibronectin Extra Domain-A and B (Fn-EDA and Fn-EDB) isoforms is associated with perturbations such as upregulation of cell proliferation, angiogenesis, and tumor metastasis [13], and Fn-EDA has been used as a target for affinity-based delivery to endometriotic lesions [12]. Due to their robust overexpression in solid tumors, TNC-C and Fn-EDB are considered promising targets for precision delivery of therapeutics and imaging agents to malignant lesions [14,15]. Recently, we reported the identification and characterization of a panel of homing peptides that target tumor ECM: a peptide that binds to TNC-C and Fn-EDB (PL1 peptide) [16], to Fn-EDB and neuropilin-1 (NRP-1) (PL2 peptide) [17], and to TNC-C and NRP-1 (PL3 peptide) [18]. Systemic PL1- and PL3-guided therapeutic nanoparticles reduced the progression of glioblastoma xenografts and increased the survival of tumor-bearing mice [16,18].

In the current study, we investigated the therapeutic potential of homing peptide-based targeting of endometriosis. Using a phage pool displaying a panel of known peptides, binding studies were carried out with cultured immortalized endometriotic cells, which demonstrated a robust binding and internalization of PL1 peptide-displaying phages. Silver nanoparticles (AgNPs) functionalized with synthetic PL1 peptide bound to and penetrated into endometriotic cells in 2D and 3D cultures. As a proof-of-concept efficacy study, we demonstrated the ability of AgNPs loaded with the antimitotic drug monomethyl auristatin E (MMAE) [19] to suppress the viability of endometriotic cells cultured in 2D and as spheroids in a peptide-dependent manner. Finally, in a cryosections overlay assay, we observed preferential accumulation of PL1-nanoparticles in Fn-EDB- and TNC-C-positive regions of human endometriosis samples.

These studies suggest a potential application for PL1-guided precision drugs, imaging agents, and nanoparticles for the detection and therapy of endometriosis.

## 2. Materials and Methods

### 2.1. Materials

Oligonucleotides were purchased from Integrated DNA Technologies IDT (Coralville, IA, USA). Biotinylated peptides (see Table 1 and Table S1) were purchased from TAG Copenhagen, Denmark. Tripotassium hexacyanoferrate(III) (K_3_Fe(CN)_6_; CAS# 13746-66-2), sodium thiosulfate pentahydrate (Na_2_S_2_O_3_; CAS# 10102-17-7), Tween-20, paraformaldehyde (PFA), Triton-X, NP40 (IGEPAL^®^ CA-630), LB medium, and 4′,6-diamidino-2-phenylindole (DAPI) were purchased from Sigma-Aldrich, Taufkirchen, Germany. IPTG was purchased from Carbosynth Limited, Compton, UK. Dulbecco’s Modified Eagle Medium (DMEM) and phosphate-buffered saline pH 7.4 (PBS) were purchased from Lonza, Verviers, Belgium. DMEM/F-12 medium with HEPES and without phenol red, McCoy’s 5A Medium with HEPES, Penicillin, Streptomycin, TrypLE Express Enzyme without phenol red, and Trypan Blue stain were purchased from Gibco, Thermo Fischer Scientific, Waltham, MA, USA. CellStripper Dissociation Reagent was purchased from Corning, Thermo Fischer Scientific, Waltham, MA, USA. Bovine serum albumin (BSA), fetal bovine serum (FBS), and charcoal stripped FBS were purchased from Capricorn Scientific, Ebsdorfergrund, Germany. Goat serum was purchased from GE Healthcare, Chalfont Saint Giles, UK. For the synthesis and functionalization of silver nanoparticles (AgNPs), AgNO_3_ (#209139), trisodium citrate hydrate (#25114), 4-morpholineethanesulfonic acid hemisodium salt (#M0164), tris(2-carboxyethyl)phosphine hydrochloride solution (TCEP; #646547), the dye CF555 succinimidyl ester (NHS-CF555, #SCJ4600022), and dimethyl sulfoxide (DMSO) were purchased from Sigma-Aldrich, Taufkirchen, Germany. NeutrAvidin (#31055) was purchased from Thermo Fischer Scientific, Waltham, MA, USA, OPSS-PEG(5K)-SCM linker from JenKem Technology, Plano, TX, USA, and lipoic acid-PEG(1K)-NH2 (#PG2-AMLA-1k) from Nanocs, Boston, MA, USA. OSu-Glu-Val-Cit-PAB-monomethyl auristatin E (NHS-MMAE-linker) was purchased from Concortis Biotherapeutics, San Diego, CA, USA.

### 2.2. Cell Culture

Human immortalized endometriotic epithelial 12Z (cat. No T0764) and human immortalized endometrial stromal HESC (cat. No T0533) cells were purchased from ABM (Richmond, BC, Canada). 12Z cells were cultured in DMEM supplemented with 10% FBS, 100 U/mL penicillin, and 100 μg/mL streptomycin. HESC cells were cultured in DMEM/F-12 medium without phenol red that was supplemented with 10% Charcoal-stripped FBS, 100 U/mL penicillin, and 100 μg/mL streptomycin. During cell passaging, CellStripper dissociation reagent was used for 12Z and TrypLE Express Enzyme without phenol red for HESC detachment. Cells were grown at 37 °C, 5% CO_2_ in a humidified environment.

### 2.3. Phage Binding to Cultured 12Z and HESC Cells

Peptide-displaying T7 bacteriophages were prepared by cloning nucleotide sequences coding for different peptides into phage genomic DNA to be expressed on the phage surface as C-terminal fusions to capsid protein. The complementary oligonucleotides were annealed, and the cloning was performed according to the manufacturer’s protocol using the T7Select^®^ 415-1 Cloning Kit (Millipore, 70015-3, Merck KGaA, Darmstadt, Germany). Peptide-displaying phage clones were thereafter amplified in complementing host bacterial cells BLT5403 and purified with polyethylene glycol according to an established protocol [24].

Prior to phage addition, 12Z and HESC cells were detached, washed with PBS, and resuspended in 0.5% BSA containing DMEM. Phages expressing a single peptide of interest (5 × 10^7^ PFU/mL) were added to 5 × 10^7^ cells, wherein insertless phage was used as a negative control. Incubation with phage clones was carried out on a slowly rocking platform at 4 °C for 1 h. Cells were washed four times with 0.5% BSA containing DMEM to eliminate unbound phages. Finally, the cells together with bound phages were resuspended in 1% NP40 containing LB solution, and plaque assay was performed as described earlier [25]. Briefly, the amount of phage in cell lysates was determined by titering using Escherichia coli strain BLT5615 in LB medium containing 2 mM IPTG.

### 2.4. Synthesis and Functionalization of AgNPs

The AgNPs were synthesized according to the citrate method of Lee and Meisel [26]. Surface functionalization of AgNPs was carried out as previously published [19,27], wherein biotinylated peptides were used as the targeting moiety, CF555 dye as the fluorophore, and MMAE-linker as a therapeutic payload. Briefly, AgNO_3_ (360 mg) was dissolved in high resistivity water (2 L; resistivity 18 MΩ cm^−1^) in a flask cleaned with piranha solution (H_2_SO_4_/H_2_O_2_). Next, trisodium citrate hydrate (400 mg) was dissolved in high resistivity water (40 mL) and added to the vessel. The solution was boiled in the dark for 30 min. The resulting Ag-citrate was used directly in the next step. Simultaneously, NeutrAvidin (NA) was modified with an OPSS-PEG(5K)-SCM linker (OPSS) according to the procedure described by Braun et al. [27]. Next, NeutrAvidin-OPSS (3.9 mL, 2.9 mg/mL) was added to the Ag-citrate solution (500 mL). After 2 min, 4-morpholineethanesulfonic acid hemisodium salt (5 mL, 0.5 M in high resistivity water) was added. The pH of the solution was adjusted to 6.0, and the solution was kept at 37 °C for 24 h. The solution was brought to room temperature (RT), and 10× phosphate-buffered saline (50 mL) was added, followed by Tween-20 (250 µL). The solution was centrifuged at 12,200× *g* at 4 °C for 1 h; the supernatant was removed, and the particles were resuspended in PBST (0.005% Tween-20 in PBS). Next, TCEP solution was added to a final concentration of 1 mM, followed by incubation at RT for 30 min. Then lipoic acid-PEG(1K)-NH_2_ was added to a final concentration of 5 µM, and the mixture was incubated at RT for 2.5 h. The solution was centrifuged at 17,200× *g* at 4 °C for 20 min; the supernatant was removed, and the particles were resuspended in PBST at half of the initial volume. The AgNP solution was filtered through a 0.45 µm filter and stored at 4 °C in dark.

NHS CF555 dye or NHS-functionalized MMAE-linker was coupled to the amino groups of the linker on the AgNPs. For this, NHS-CF555 or NHS-MMAE-linker (5 µL, 2 mM) in DMSO was added to AgNPs (500 µL), followed by incubation at 4 °C overnight. The particles were washed 3 times by centrifugation at 3500× *g* at 4 °C for 10 min, followed by resuspension of the particles in PBST by sonication. Next, biotinylated peptides were coupled to the particles by adding the peptide (10 µL, 2 mM in high resistivity water) to AgNPs (500 µL), followed by incubation at RT for 30 min. The AgNPs were washed, 0.2 µm filtered and stored in the dark at 4 °C.

### 2.5. Characterization of AgNPs

For transmission electron microscopy (TEM), the AgNPs were diluted in MQ water, dropped onto the carbon film-covered side of a copper Cu-300 TEM grids (Agar Scientific, Ltd., Essex, UK), air-dried, and imaged with a Tecnai-10 transmission electron microscope (FEI Company, Hillsboro, OR, USA) at 80 kV. The concentration of AgNPs was calculated according to the Beer–Lambert law by measuring the UV–Vis absorbance of AgNPs at 415 nm and using a molar attenuation coefficient of 8.83 × 10^−9^ M^−1^ cm^−1^. For size and zeta potential measurements, the AgNPs were diluted 400X in MQ water. Zetasizer Pro (Malvern Panalytical, Malvern, UK) was used for the measurements. Size measurements were done at 25 °C with the material refractive index (RI) set to 2.00, and material absorption set to 0.200 using polystyrene cuvettes (#DTS0012, Malvern Panalytical, Malvern, UK); zeta potential measurements were done at 25 °C with 12 runs/measurement using capillary cell cuvettes (#DTS1070, Malvern Panalytical, Malvern, UK).

### 2.6. Expression Analysis of Peptide Receptors in Cultured 12Z and HESC Cells

The expression of peptide receptors on 2D cultured cells was studied using immunofluorescence staining. 12Z and HESC cells (100,000 cells) were seeded onto noncoated coverslips (12 mm diameter, Marienfeld-Superior, Paul Marienfeld GmbH & Co.KG, Lauda-Königshofen, Germany) in a 24-well plate and cultured for 24 h. The cells were washed with PBS, fixed with 4% PFA in PBS for 10 min, and incubated with a blocking buffer (5% BSA, 5% FBS, and 5% goat serum in PBS) at RT for 30 min. The cells were incubated with a solution of rabbit polyclonal anti-TNC-C (15 µg/mL), rabbit polyclonal anti-Fn-EDB (20 µg/mL), mouse monoclonal anti-NRP-1 (6 µg/mL) [16,18], or mouse anti-integrin αv [272-17E6] (ab16821, Abcam) (5 µg/mL) antibodies in blocking buffer diluted 1:5 in PBS at RT for 30 min. After washes with PBS, the cells were incubated with Alexa Fluor^®^647-goat anti-rabbit IgG (A21245, Invitrogen, Thermo Fisher Scientific, Waltham, MA, USA) or Alexa Fluor^®^647 goat anti-mouse IgG (A21235, Life Technologies, Thermo Fisher Scientific, Waltham, MA, USA) (4 µg/mL in blocking buffer diluted 1:5) at RT for 30 min. Cells were stained with DAPI (2 µg/mL) for 10 min, mounted with mounting medium (Fluoromount-G; Electron Microscopy Sciences, Hatfield, PA, USA) and visualized using a confocal microscope FV1200MPE (Olympus, Shinjuku, Japan) equipped with a UPlanSApo 60x/1.35na objective (Olympus, Shinjuku, Japan). The images were analyzed using Olympus FluoView Ver.4.2a Viewer software.

### 2.7. Luminescence-Based Cellular Viability Assay

12Z and HESC cells (10,000 cells) were cultured in white 96-well plates for 24 h. Cells were treated with different concentrations of PL1-MMAE-AgNPs or biotin-MMAE-AgNPs at 37 °C for 2 h and washed with full medium. Cells in fresh medium were cultured for an additional 48 or 72 h, and the cell viability was measured using the CellTiter-Glo^®^ Luminescent Cell Viability Assay (#G7570, Promega Biotech AB, Madison, WI, USA) following the manufacturer’s protocol. Luminescence was measured with a Victor X5 Multilabel Microplate Reader (Perkin Elmer, Waltham, MA, USA). The cell viability was calculated as % viability = (bioluminescence of the sample) × 100/(bioluminescence of cells incubated only with the medium).

### 2.8. AgNP Interaction with Cells in 2D and 3D Cultures

To study AgNP uptake in 2D cell culture, 12Z and HESC cells (100,000 cells) were seeded in 24-well plates and cultivated for 24 h. Subsequently, the cells were incubated with CF555-labeled AgNPs (0.3 nM in DMEM with 10% FBS) at 37 °C for 2 h, washed, treated with an etching solution (1 mM of K_3_Fe(CN)_6_ and Na_2_S_2_O_3_ in PBS) at RT for 5 min, washed with PBS, and detached with non-enzymatic cell dissociation buffer (Corning™ CellStripper Dissociation Reagent, Thermo Fisher Scientific, Waltham, MA, USA). Cells in suspension were collected by centrifugation at 300× *g* for 5 min, resuspended in 0.2 mL PBS and analyzed by flow cytometry (BD Accuri C6 Plus, BD Biosciences, Franklin Lakes, NJ, USA). The data analysis was done using the same software.

For AgNP uptake and penetration studies in 3D cultures, 12Z cell spheroids were formed by using the magnetic 3D bioprinting method [28]. Briefly, 12Z cells at 70% confluency in a 6-well plate were loaded with magnetic nanoparticles (Nanoshuttle, n3D Biosciences, Houston, TX, USA) in a humidified atmosphere containing 5% CO_2_ at 37 °C for 8 h. Cells were trypsinized, centrifuged, and seeded into ultra-low attachment 96-well plate in a volume of 100 µL (2000 cells/well). The plate was placed on a magnetic drive and incubated in a humidified atmosphere with 5% CO_2_ at 37 °C for 2 days. Subsequently, the spheroids were incubated with 0.3 nM of fluorescently labeled AgNPs for 1, 2, 4, or 20 h. To remove extracellular particles, spheroids were treated with the etching solution, washed twice with PBS, fixed in 4% buffered PFA, and cell nuclei were counterstained with DAPI. The AgNPs in spheroids were visualized using a confocal laser scanning microscope (FluoView FV1000, Olympus, Shinjuku, Japan; 10× objective).

### 2.9. Treatment of 12Z Spheroids with Cytotoxic AgNPs

For the morphological studies, 12Z spheroids were cultured in the presence of 0.6 nM PL1-MMAE-AgNPs or biotin-MMAE-AgNPs (60 nM of MMAE) in complete medium in a humidified atmosphere with 5% CO_2_ at 37 °C. The effect of the treatment on the spheroid morphology was studied every 24 h using the Olympus IX73 inverted microscope (Olympus, Shinjuku, Japan).

For the quantitative viability studies, 12Z spheroids were incubated with different concentrations of PL1-MMAE-AgNPs or biotin-MMAE-AgNPs for 72 h, and the number of live cells was determined with a luminescence-based cell viability kit (CellTiter-Glo^®^, Promega, Madison, WI, USA), according to the manufacturer’s protocol. The luminescence was measured using a Tecan Infinite 200 microplate reader (Tecan, Männedorf, Switzerland). Cell viability in spheroids was calculated as % viability = (bioluminescence of the sample) × 100/(bioluminescence of cells incubated only with the medium).

### 2.10. Clinical Endometriosis Sample Collection and Immunostaining

Endometrial and endometriotic lesion tissues from the patient were collected during laparoscopy at the Tartu University Hospital Women’s Clinic. The patient was of reproductive age, 28 years old, had not received any hormonal medication three months before the recruitment, and was suffering from stage IV endometriosis. Sample collection procedure was approved by the Research Ethics Committee of the University of Tartu (approval No. 276/M-13, Tartu, Estonia), and informed written consent was obtained from the participant.

Following resection, endometrial and peritoneal endometriosis samples were transferred to cold McCoy’s 5A Medium. Tissues were snap-frozen in liquid nitrogen and stored in a −80 °C freezer. Prior to cryosectioning with a Leica CM1520 cryostat, the tissues were embedded in Tissue Freezing Medium (Leica Biosystems, Wetzlar, Germany). Tissue sections (10 µm) were collected on SuperFrost Plus slides (Thermo Fischer Scientific, Waltham, MA, USA) and stored at −20 °C.

For immunofluorescence staining and AgNP binding experiments, sections were air-dried at RT for 20 min followed by re-hydration in cold PBS. Sections were fixed in ice-cold absolute methanol for 10 min, washed with PBS, and permeabilized with 0.2% Triton-X in PBS for 10 min. After washing with PBST (0.05% Tween-20 in PBS), sections were incubated with 5% blocking buffer in PBST at RT for 1 h.

For immunofluorescence, the tissues were incubated with rabbit polyclonal anti-TNC-C (30 µg/mL) or rabbit polyclonal anti-Fn-EDB (40 µg/mL) primary antibodies in blocking buffer diluted 1:5 in PBST at RT for 1 h. After washes with PBST, tissues were incubated with Alexa Fluor^®^647-goat anti-rabbit IgG (A21245, Invitrogen, Thermo Fisher Scientific, Waltham, MA, USA) (10 µg/mL in blocking buffer diluted 1:5) at RT for 30 min.

For AgNP binding, sections were incubated with CF555-labeled AgNPs (1.5 nM in blocking buffer diluted 1:5) at RT for 2 h. Tissues were washed with PBST and nuclei were counterstained with DAPI (2 µg/mL) for 10 min. The sections were mounted with mounting medium and visualized using a confocal microscope FV1200MPE (Olympus, Shinjuku, Japan) equipped with a UPlanSApo 10×/0.4na objective (Olympus, Shinjuku, Japan). The images were analyzed with Olympus FluoView Ver.4.2a Viewer software.

For immunohistochemistry, sections were air-dried at RT for 20 min followed by fixation in 4% PFA in PBS for 10 min, washed in PBS, and processed in the automated tissue staining system Ventana Benchmark Ultra (Roche, Ventana Medical Systems, Tucson, AZ, USA). The sections were stained with anti-CD10 (CM129C, Clone 56C6; Biocare Medical, Concord, CA, USA) and anti-CD31 (M0823, Clone JC70A; Dako, Agilent Technologies, Santa Clara, CA, USA) antibodies. Both antibodies were diluted 1:50 in Dako Real Antibody Diluent (Agilent Technologies, Santa Clara, CA, USA) and incubated at 36 °C for 32 min or 36 min with anti-CD10 or anti-CD31, respectively. OptiView DAB (Roche, Ventana Medical Systems, Tucson, AZ, USA) detection kit was used according to the manufacturer’s guidelines for CD10, and Ultraview DAB (Roche, Ventana Medical Systems, Tucson, AZ, USA) for CD31 antibody visualization.

Hematoxylin and eosin staining was conducted on the Leica ST5020 automated tissue staining system (Leica Biosystems, Wetzlar, Germany) by using the ST Infinity H&E Staining System Kit (Leica Biosystems, Wetzlar, Germany) according to the manufacturer’s protocol.

### 2.11. Statistical Analysis

Statistical analysis was performed with Statistica 8 software (StatSoft Europe, Hamburg, Germany) using Fisher LSD and the one-way ANOVA tests. The significance level was set at *p* < 0.05.

## 3. Results and Discussion

### 3.1. TNC-C and Fn-EDB Targeting Peptide-Displaying Phages Bind to 12Z and HESC Cells

Peptide phage biopanning has been used for identification of a multitude of peptides able to recognize differentially expressed vascular markers in healthy tissues and at sites of disease (e.g., malignant, inflammatory, atherosclerotic and neurodegenerative lesions) [24,29,30]. To map the landscape of homing peptides potentially useful for targeting endometriotic lesions, we first studied the binding of a panel of T7 phages displaying different tumor homing peptides to two cultured human cell lines which are commonly used as models of endometriotic cells: 12Z (immortalized epithelial-like ectopic endometriotic cells) and HESC (immortalized eutopic endometrial stromal cells) cells [31,32]. These cell lines were chosen to represent the stromal and the glandular epithelial compartments of the endometrium, both known to play a role in the pathogenesis of endometriosis. Endometriosis, like cancer, is an angiogenesis-dependent disease, and increased vasculature and newly formed blood vessels are often observed in areas surrounding endometriotic lesions [33]. Factors involved in the promotion of vascular growth and permeability, such as the vascular endothelial growth factor (VEGF), bradykinin, and reactive oxygen species, are also associated with endometriosis [33,34,35]. As neovascularization is a shared hallmark in the pathogenesis of both endometriosis and cancer, we included in the binding studies the homing peptides that target molecules expressed in angiogenic vessels: PL1 (receptors: TNC-C and Fn-EDB), PL2 (Fn-EDB and NRP-1), PL3 (TNC-C and NRP-1), prototypic C-end Rule (CendR) peptide RPARPAR, referred from now as RPAR (NRP-1), and iRGD peptide (angiogenic αv integrins and NRP-1) (Table 1). In addition, we added phages displaying two peptides reported to bind endometriotic lesions (VRRADNRPG and RTRLHTR that we refer to as EM1 and EM2 peptides) (Table 1) and insertless phage as a negative control.

**Figure 1:**
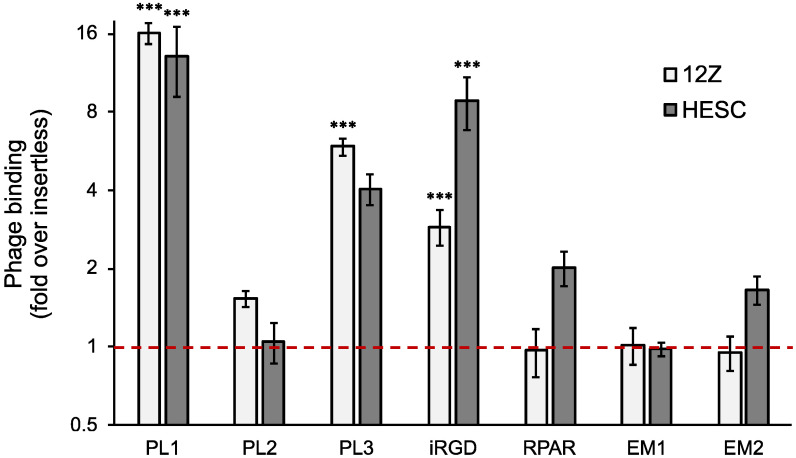
Binding of peptide-phages to cultured 12Z and HESC cells. Cells were incubated with the different peptide-displaying phages at 4 °C for 1 h, washed, and phage binding quantified by plaque assay. The phage binding is represented as a fold over insertless phage. N = 3, error bars = ±SEM. *** *p* < 0.001 (compared with insertless phage).

The phages displaying bispecific TNC-C and Fn-EDB targeting PL1 peptide showed the strongest binding to both HESC and 12Z cells (13–16-fold over the insertless phage) (Figure 1). The phages displaying another TNC-C targeting peptide, PL3, also bound to both HESC and 12Z cells (4–6-fold over the insertless phage), followed by the phages displaying iRGD peptide (3- and 9-fold over the insertless for 12Z or HESC, respectively). Only low/background binding to 12Z cells was seen for the RPAR, EM1, and EM2 phages, and RPAR and EM2 showed modest binding to HESC cells (~2-fold over the insertless). EM1 peptide (z13) was identified by in vivo phage display on mouse peritoneal cavity and Ishikawa cells [22]. The receptor for z13 is the cyclic nucleotide-gated channel β3 (CNGB3), a sorting pathway protein highly expressed in endometrial glandular epithelial cells and peritoneal surfaces in clinical endometriosis samples. EM2 peptide was identified by phage biopanning on clinical endometriosis samples and was shown to bind to ectopic endometrial stromal cells [23]. It is possible that the receptors for these peptides are not upregulated in the cell culture system used here, or the EM1 and EM2 peptides are context-dependent, and some aspects of the T7 peptide display system used here (e.g., C-terminal exposure, peptide density of about 200 peptides per 55 nm T7 particle, or linker) are not suited to reveal the efficient binding to the target using the protocols of the current study.

### 3.2. PL1 Receptors Are Expressed in Cultured 12Z and HESC Cells

**Figure 2:**
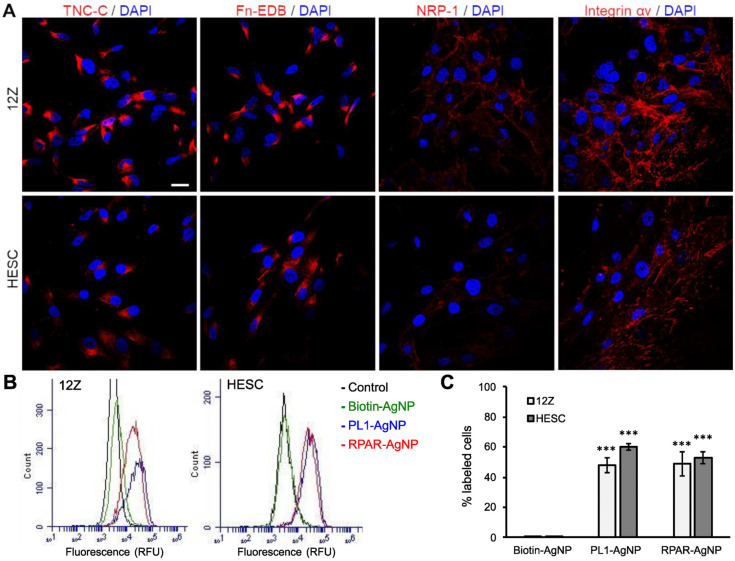
Peptide receptor expression and peptide-nanoparticle internalization in 12Z and HESC cells. (**A**) Expression of Fn-EDB, TNC-C, and cell surface NRP-1 and integrin αv in attached 12Z and HESC cells. Attached cells were fixed; incubated with anti-Fn-EDB, anti-TNC-C, anti-NRP-1, or anti-integrin αv antibodies; and stained with the secondary fluorescent antibodies. Red: Fn-EDB, TNC-C, NRP-1, integrin αv. Blue: DAPI. Scale bar = 20 µm. (**B**,**C**) Cell internalization of peptide-AgNPs. Attached cells were incubated with the fluorescently labeled peptide-AgNPs at 37 °C for 2 h, treated with the etching solution and detached, and the fluorescence was measured by flow cytometry. N ˃ 4. Error bars = ±SEM. Panel B shows the number of cells (counts) vs. mean fluorescence. Panel C shows the % of fluorescent cells after incubation with the AgNPs. *** *p* < 0.001 (compared with control biotin-AgNPs).

The expression of the receptors recognized by the homing peptides (TNC-C, Fn-EDB, NRP-1, and integrin αv) in 12Z and HESC cells was studied by confocal microscopy imaging. Confocal microscopy demonstrated that both cells expressed TNC-C and Fn-EDB, αv integrins, and, to a lesser extent, NRP-1 (Figure 2A). The peptide receptor expression correlated with the peptide-phage binding, with the highest cell binding observed for the ECM targeting peptides, followed by iRGD (integrin αvβ_3/5_ ligand) and RPAR (binding to NRP-1), which is in line with the data on the peptide-displaying phage binding to the cells (Figure 1).

### 3.3. PL1-Targeted Nanoparticles Internalize in 12Z and HESC Cells

In contrast to mono-specific targeting ligands, PL1 engages with two target molecules, Fn-EDB and TNC-C; this engagement is primarily driven by electrostatic interactions [36]. Simultaneous targeting of two ECM receptors “averages” the accumulation of peptide and its payload over target tissue resulting in more uniform distribution. We have recently shown that PL1 not only binds to the tumor ECM but also internalizes in malignant cells by macropinocytosis [36]. This feature provides an important advantage over targeting ligands that are reported to be non-internalizing. Firstly, cellular uptake increases the accumulation of the PL1 peptide and its payloads; secondly, cell internalization can dramatically expand the range of PL1 therapeutic payloads to intracellularly-acting cytotoxic agents and radionuclides.

We next tested whether PL1-functionalized nanoparticles are internalized in cultured 12Z and HESC cells. AgNPs with a size of around 100 nm and Z-potential of −18 mV (Figure S1) were functionalized with PL1 peptide (PL1-AgNPs), and the cellular entry was tested in both cells. Internalized and non-internalized AgNPs can be distinguished by treating the cells with a biocompatible etching solution that eliminates the extracellular AgNPs [27]. Several studies show that nanoparticles of different core composition (Au, Cd, polymers, lipids, etc.), size range (10–150 nm), and surface charge (+30–−20 mV) accumulate in endometriotic tissue [37]. The AgNPs that we used here share some features (size ~100 nm, surface PEGylation, and negative Z-potential) with clinically used nanoparticles, such as Caelyx, that show enhanced tumor accumulation and long plasma half-life. However, our previous studies demonstrated that peptide-functionalization significantly increased the tumor homing of nanoparticles [16,38,39,40,41,42,43,44,45,46,47,48,49], and therefore, PL1-functionalization might also increase the accumulation of the AgNPs in endometriotic lesions. Biotinylated peptides were coated on neutravidin-functionalized fluorescently labeled AgNPs, and biotin-blocked AgNPs (biotin-AgNPs) were used as a negative control. As both cell lines express NRP-1, a cellular recruitment and internalization receptor of CendR peptides, we also included RPAR as a positive control and a representative of a monospecific peptide that engages with the NRP-1. Moreover, RPAR is also positively charged like PL1. After incubation with AgNPs, the cells were treated with the etching solution, and the remaining cell-bound fluorescence was quantified by flow cytometry. After incubation for 2 h, PL1-AgNPs were internalized in both cell lines (Figure 2B,C), whereas the cells incubated with control biotin-AgNPs remained negative. RPAR-functionalized AgNPs (RPAR-AgNPs) were also internalized in both cell lines, supporting the ability of NRP-1 to take up CendR cargoes. These results show that PL1 and CendR peptides promote the internalization of nanoparticles in 12Z and HESC cells via interaction with proteins of the ECM and the NRP-1 receptor, respectively.

### 3.4. PL1 Conjugation Increases the Effect of Cytotoxic Nanoparticles in 12Z and HESC Cells

Several nanoparticle-based modalities have been developed as nonhormonal therapies for endometriosis, including gene therapy [50,51,52], phototherapy [53,54], immunotherapy [55], and combinational therapy [56]. We have also recently demonstrated cell-penetrating peptide and siRNA-mediated therapeutic effects on endometriosis models and proposed a novel therapeutic target, ribonucleotide reductase for the treatment of endometriosis [57]. In addition, we have previously identified miRNAs that are differentially expressed in ectopic endometriotic stromal cells [58] and found that some anticancer drugs, such as doxorubicin, are selectively cytotoxic to ectopic endometrial cells [59]. Affinity-targeting of such drugs using nanoformulations could be an efficacious strategy to potentiate their activity of specifically depleting endometriotic lesions.

**Figure 3:**
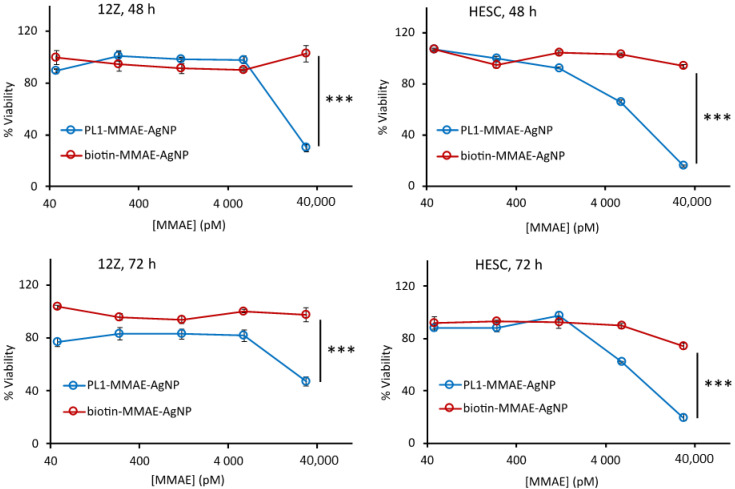
PL1 conjugation increases the cytotoxicity of MMAE-AgNPs. Attached 12Z and HESC cells were incubated with different concentrations of PL1-MMAE-AgNPs or biotin-MMAE-NPs at 37 °C for 2 h, washed, and incubated for an additional 48 or 72 h. The cytotoxicity was measured using the CellTiter-Glo^®^ viability assay. The cytotoxicity is represented as a percentage of viability relative to the untreated cells. N = 5, error bars = ±SEM, *** *p* ˂ 0.001.

Next, we investigated the cytotoxic effect of PL1-AgNPs loaded with the potent antimitotic agent MMAE (PL1-MMAE-AgNPs) [19]. MMAE was loaded onto the AgNPs via a cathepsin B-cleavable valine-citrulline linker. We tested a range of MMAE concentrations to evaluate the peptide-dependent cytotoxicity in 12Z and HESC cells. We tested a range of MMAE concentrations to evaluate the peptide-dependent cytotoxicity in 12Z and HESC cells. The cells were incubated with PL1-MMAE-AgNPs or with the untargeted biotin-MMAE-AgNPs at 37 °C for 2 h, washed to remove unbound AgNPs, and incubated for an additional 48 or 72 h (Figure 3). At the highest MMAE concentration (30 nM), the treatment with PL1-MMAE-AgNP resulted in a ~50% decrease in the number of viable 12Z cells and in a ~80% decrease of viable HESC cells. HESC cells appeared more sensitive to the treatment and showed ~35% decrease in viability also at a 6 nM MMAE concentration. In contrast, at the same MMAE concentrations, negative control biotin-MMAE-AgNPs did not have a significant effect on the cell viability. The higher cytotoxic effect observed in HESC compared with 12Z cells could be due to the different chemosensitivity of endometriotic epithelial and stromal cells, similar to differential chemoresponsiveness observed between malignant epithelial and stromal cells of malignant carcinomas [60]. These results show that the PL1-guided therapeutic nanoparticles are able to efficiently deliver an intracellularly-acting drug inside the 12Z and HESC cells.

To bind MMAE to AgNPs we used a valine-citrulline linker that is cathepsin B sensitive. Some ECM proteases, such as matrix metalloproteinases, are overexpressed in ectopic endometrial tissue [61,62]. It is possible that application of alternative enzyme-cleavable linkers might result in increased release of the drug in endometriotic lesions as we have shown previously in tumor mouse models [63,64].

### 3.5. PL1 Promotes the Penetration and Cytotoxicity of AgNPs in Endometriotic Spheroids

We next studied the cellular internalization and penetration of PL1-AgNPs into 12Z spheroids. Three-dimensional cell culture mimics the properties of tissues better than the regular monolayer cultures [65]. Compared to 12Z epithelial cells grown as 2D monolayers, 3D 12Z spheroids are morphologically and functionally more similar to the actual clinical lesions [66].

**Figure 4:**
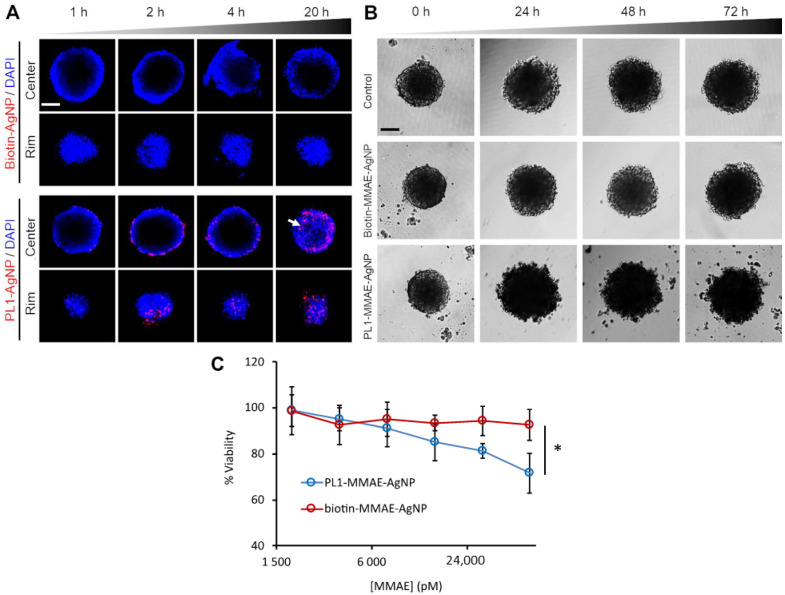
PL1 conjugation increases the penetration and internalization of AgNPs in 12Z spheroids and the cytotoxicity of MMAE-AgNPs. (**A**) Penetration and internalization of PL1-AgNPs in 12Z spheroids. The spheroids were incubated with fluorescently labeled PL1-AgNPs or biotin-AgNPs for 1, 2, 4, and 20 h, and the internalized AgNPs were detected by confocal fluorescence microscopy. Red = AgNPs; Blue = DAPI. Scale bar = 100 µm. (**B**) Morphology of spheroids after incubation with PL1-MMAE-NP or biotin-MMAE-AgNPs. Representative images of 12Z spheroids incubated for 24, 48, and 72 h with the AgNPs. Scale bar = 100 µm. (**C**) Toxicity of PL1-MMAE-AgNPs in 12Z spheroids. Spheroids were incubated with different concentrations of PL1-MMAE-AgNPs or biotin-MMAE-AgNPs for 72 h, and the cell viability was measured by the CellTiter-Glo^®^ viability assay. N = 3. Error bars = ±SEM. * *p* ˂ 0.05.

The spheroids were incubated with fluorescent PL1-AgNPs or nontargeted AgNPs (biotin-AgNPs) for different times, treated with the etching solution to remove exposed AgNPs, and imaged by confocal fluorescence microscopy. Microscopic imaging of the central core and peripheral rim of the spheroids showed that at 2 and 4 h of incubation, the PL1-AgNPs were internalized in the spheroid cells at the periphery of the spheroids (Figure 4A). After 20 h of incubation, PL1-AgNPs were also found deeper in the spheroids (Figure 4A, white arrow). In contrast, biotin-AgNPs did not bind and penetrate the spheroids.

Next, the effect of PL1-MMAE-AgNPs on the integrity and viability of 12Z spheroids was evaluated. The integrity of the spheroids treated with PL1-MMAE-AgNPs was compromised already after 24 h of incubation. The damage, seen as loss of the compact structure and blebbing, became more pronounced after 48 h (Figure 4B). In contrast, the untreated spheroids or spheroids treated with biotin-MMAE-AgNPs showed no damage and continued growing until the 72 h time point. The viability of 12Z cells in 3D culture was also tested at 72 h of incubation with different concentrations of PL1- and biotin-MMAE-AgNPs. The quantitation of the viable cells in spheroids after 72 h treatment showed that in comparison to spheroids treated with biotin-MMAE-AgNPs, PL1-MMAE-AgNP-treated spheroids had ~20% decrease in the number of viable cells at 60 nM of drug (Figure 4C). These data show that PL1 induces the internalization and cytotoxic effect of nanoparticles in 3D culture of endometriotic cells.

### 3.6. PL1-AgNPs Bind to TNC-C- and Fn-EDB-Positive Areas of Human Peritoneal Endometriotic Lesions

**Figure 5:**
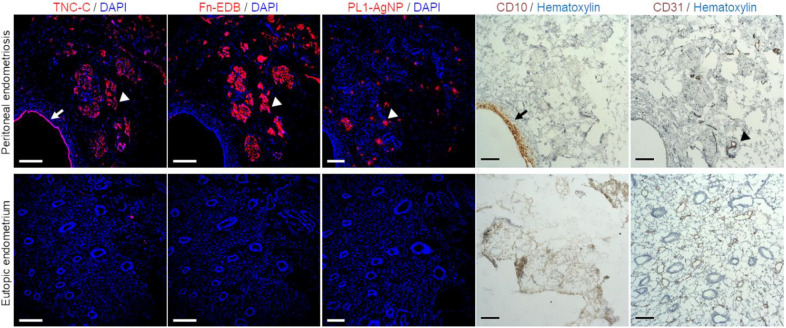
Clinical peritoneal endometriotic lesions express TNC-C and Fn-EDB, and PL1-AgNPs bind to the endometriotic lesions. Human peritoneal endometriotic lesion and human eutopic endometrium samples were cryosectioned and immunostained with Fn-EDB, TNC-C, CD10, and CD31 or incubated with fluorescently labeled PL1-AgNPs or control AgNPs. The control AgNPs binding is shown in Figure S2. Red: TNC-C (white arrow), Fn-EDB (white arrowhead), PL1-AgNP (white arrowhead); brown: CD10 (black arrow), CD31 (black arrowhead); blue: DAPI or hematoxylin. Scale bar = 150 µm.

To assess the translational potential of PL1-mediated targeting, the ex vivo binding of PL1-AgNPs and the expression of PL1 peptide’s receptors TNC-C and Fn-EDB were evaluated in clinical peritoneal endometriosis samples. First, the endometriotic lesion sites in the clinical sample were mapped by immunohistochemistry. The staining for CD10, a marker of ectopic endometrial stroma [67], demonstrated the presence of a large endometriotic gland surrounded by stroma in the peritoneal tissue (Figure 5, black arrow). TNC-C immunoreactivity localized to the epithelial cells of the endometriotic gland (Figure 5, white arrow) but not to the glands of eutopic endometrium. Fn-EDB was also detected in the peritoneal endometriosis sample but not in the eutopic endometrium (Figure 5, white arrowhead), indicating a differential TNC-C and Fn-EDB expression in ectopic and eutopic endometrial tissues. TNC-C and Fn-EDB signal was observed in areas around the CD31-positive blood vessels (Figure 5, black arrowhead). After incubating the cryosections with PL1-AgNPs and washing, we observed nanoparticles in endometriotic lesions in the perivascular areas positive for the expression of TNC-C and Fn-EDB (Figure 5, white arrowheads), while no signal was seen in the eutopic endometrium. This result suggests that PL1 targets not only ectopic endometriotic cells but also neovessels surrounding the endometriotic lesions, thus further demonstrating the advantage of using PL1-AgNPs as a theranostic tool.

## 4. Conclusions

PL1 homing peptide has the potential to be positioned as an enabling technology both in endometriosis diagnostics (e.g., guided surgery, imaging) as well as therapeutics, expanding the therapeutic index of drugs by maximizing efficacy and limiting systemic side effects. Our results encourage further studies for their preclinical development. The in vivo homing of the PL1-targeted nanoparticles to endometriosis lesions and their therapeutic effect in animal models as well as the exploration of different therapeutic and imaging cargoes will be the subjects of follow-up studies.

## Figures and Tables

**Table 1:** Sequences of the peptides used. * Disulfide bond.

Peptide ID	Sequence	Receptor	Reference
PL1	PPRRGLIKLKTS	TNC-C and Fn-EDB	[16]
PL2	TSKQNSR	Fn-EDB and NRP-1	[17]
PL3	AGRGRLVR	TNC-C and NRP-1	[18]
RPAR	RPARPAR	NRP-1	[20]
iRGD	C * RGDKGPDC *	Integrin αvβ_3/5_ and NRP1 after cleavage	[21]
EM1	VRRADNRPG	Cyclic nucleotide-gated channel β3 (CNGB3)	[22]
EM2	RTRLHTR	Unknown	[23]

## Data Availability

The datasets used and/or analyzed during the current study are available from the corresponding author on reasonable request.

## Supplementary Materials

The following are available online at https://www.mdpi.com/article/10.3390/nano11123257/s1, Figure S1. Characterization of AgNPs. Table S1. Synthetic peptides used for nanoparticle functionalization. Figure S2. Control biotin-AgNPs do not bind to clinical endometriotic lesions.
