# Supplementary material for: Homing Peptide-Based Targeting of Tenascin-C and Fibronectin in Endometriosis
Source: Nanomaterials (Basel). 2021 Nov 30;11(12):3257. doi: 10.3390/nano11123257 (PMC8708492; doi:10.3390/nano11123257)
Supplement: Supplementary file 1 [file nanomaterials-11-03257-s001.zip › nanomaterials-1450141-supplementary.pdf]

nanomaterials

MDPI

Supplementary Materials

Homing Peptide-Based Targeting of Tenascin-C and Fibronectin in Endometriosis

Lorena Simón-Gracia [1,†], Kristina Kiisholts [2,†], Vilma Petrikaitė [3,4], Allan Tobi [1], Merli Saare [2,5], Prakash Lingasamy [1], Maire Peters [2,5], Andres Salumets [2,5,6,7] and Tambet Teesalu [1,8,*]

1 Laboratory of Precision and Nanomedicine, Department of Biomedicine and Translational Medicine, University of Tartu, 50411 Tartu, Estonia; Lorena.Simon.Gracia@ut.ee (L.S.-G.); allan.tobi@ut.ee (A.T.); prakash.lingasamy@ut.ee (P.L.)
2 Competence Centre on Health Technologies, 50411 Tartu, Estonia; kristina.kiisholts@ut.ee (K.K.); merli.saare@ut.ee (M.S.); maire.peters@ut.ee (M.P.); Andres.Salumets@ut.ee (A.S.)
3 Laboratory of Drug Target Histopathology, Institute of Cardiology, Lithuanian University of Health Sciences, 44307 Kaunas, Lithuania; vilmapetrikaite@gmail.com
4 Life Sciences Center, Institute of Biotechnology, Vilnius University, 10257 Vilnius, Lithuania
5 Department of Obstetrics and Gynecology, Institute of Clinical Medicine, University of Tartu, 50406 Tartu, Estonia
6 Institute of Genomics, University of Tartu, 51010 Tartu, Estonia
7 Division of Obstetrics and Gynecology, Department of Clinical Science, Intervention and Technology (CLINTEC), Karolinska Institutet, 14152 Stockholm, Sweden
8 Center for Nanomedicine, Department of Cell, Molecular and Developmental Biology, University of California at Santa Barbara, Santa Barbara, CA 93106, USA
* Correspondence: tambet.teesalu@ut.ee
† Both authors contributed equally to this work.

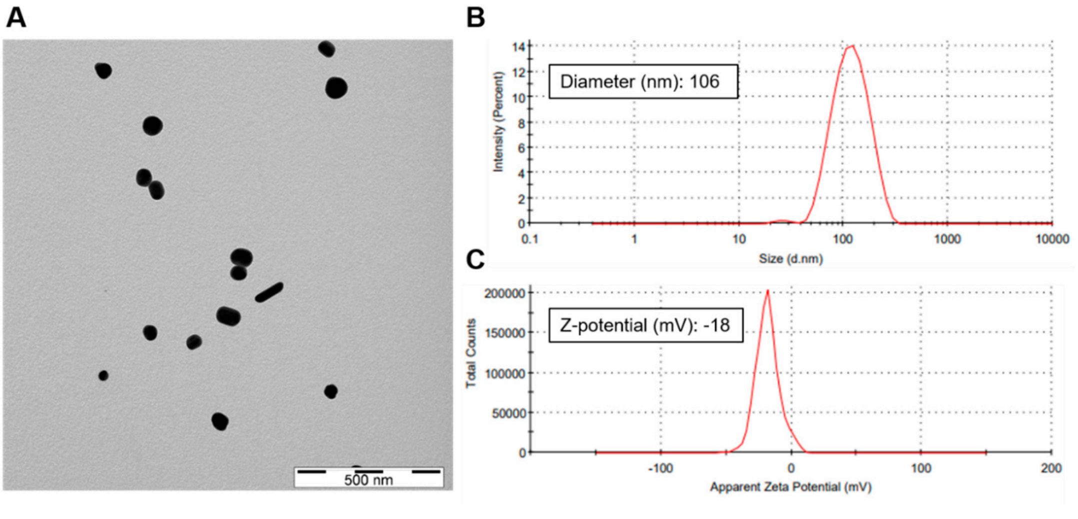

Figure S1. Characterization of AgNPs. (**A**) Transmission electron microscopy (TEM) picture of the AgNPs. (**B**) Hydrodynamic diameter of the AgNPs measured by dynamic light scattering (DLS). (**C**) Z-potential of the AgNPs.

Table S1. Synthetic peptides used for nanoparticle functionalization. Ahx = aminohexanoic acid.

Peptide ID	Sequence	Receptor
Biotin-PL1	Biotin-Ahx-PPRRGLIKLKTS	TNC-C and Fn-EDB
Biotin-RPAR	Biotin-Ahx-RPARPAR	NRP-1

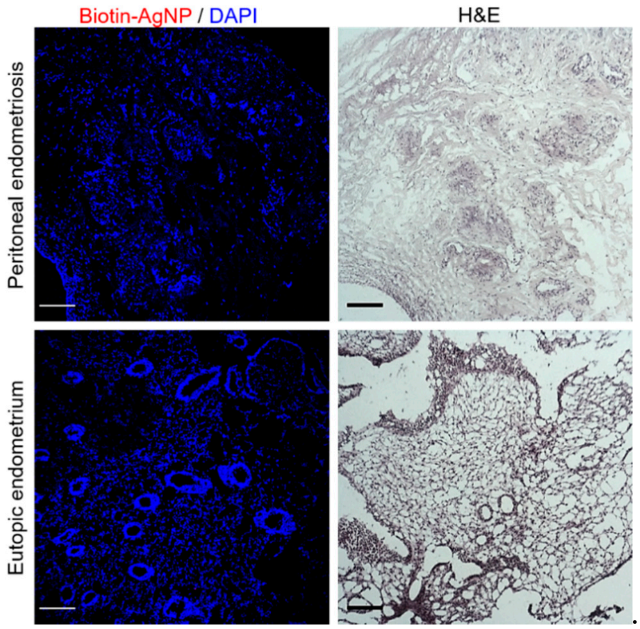

Figure S2. Control biotin-AgNPs do not bind to clinical endometriotic lesions. Human peritoneal endometriotic lesions and human eutopic endometrium samples were sectioned and the tissue slides were incubated with fluorescently labeled biotin-AgNPs or H&E stained. No signal of biotin-AgNPs was observed in either peritoneal lesions or eutopic endometrium. Red: biotin-AgNPs; blue: DAPI. Scale bar = 150 μm.
